# Support for Older Parents in Need in Europe: The Role of the Social Network and of Individual and Relational Characteristics

**DOI:** 10.1093/geroni/igad032

**Published:** 2023-04-17

**Authors:** Liora Cohen, Sharon Shiovitz-Ezra, Bracha Erlich

**Affiliations:** Israel Gerontological Data Center (IGDC), The Hebrew University of Jerusalem, Jerusalem, Israel; Israel Gerontological Data Center (IGDC), The Hebrew University of Jerusalem, Jerusalem, Israel; Paul Baerwald School of Social Work and Social Welfare, The Hebrew University of Jerusalem, Jerusalem, Israel; Israel Gerontological Data Center (IGDC), The Hebrew University of Jerusalem, Jerusalem, Israel

**Keywords:** Informal care, Child–parent relationship, SHARE

## Abstract

**Background and Objectives:**

Adult children form the backbone of informal care for older parents. To date, limited attention has been paid to the complex mechanism of providing support to older parents. The present study investigated mezzo- and micro-level correlates of provision of support to older parents. The focus was on the child–parent relationship in childhood and in the present.

**Research Design and Methods:**

Data were derived from the Survey of Health, Ageing and Retirement in Europe (SHARE). The analytic sample comprised respondents who participated in SHARE Waves 6–8 and reported having an unhealthy mother (*N* = 1,554) or father (*N* = 478). We used hierarchical logistic regression to address 3 models including individual resources, child–parent characteristics, and social resources. We conducted separate analyses for mothers and fathers.

**Results:**

Providing support to a parent depended primarily on personal resources followed by the quality of the relationship with the parent. A larger social network of the care provider was also related to increased likelihood of providing support. Support to a mother was associated with positive evaluations of the relationship with her in the present and in childhood. At the same time, negative evaluations of the relationship with the father in childhood were negatively related to providing support to him.

**Discussion and Implications:**

The findings point to a multidimensional mechanism, in which adult children’s resources are a prominent factor in shaping caregiving behaviors toward their parents. Clinical efforts should focus on adult children’s social resources and the quality of the child–parent relationship.

**Translational Significance:** The present study addresses multidimensional correlates of providing informal support to older parents. We found that providing support to a parent depended on adult children’s individual and social resources and whether it was the mother or the father who was in need of support. The quality of the child–parent relationships at present and in childhood and the size of the social network were associated with the provision of informal care. Findings underscore the importance of social policy to increase individual resources, such as financial capacity and support clinical interventions, to improve the quality of the relationship with unhealthy parents at present and resolve conflictual family issues from the past.

## Background and Objectives

The increase in life expectancy followed by higher rates of disability and dependence has led to a rise in demands for the care of older adults (Colombo et al., 2011). Currently, in Europe, informal care forms the backbone of long-term care for older adults. Across European countries, informal care for older people is estimated to range between 20% and 44% (Verbakel, 2018). Adult children are one of the main sources of informal care (de Klerk et al., 2021; Jacobs et al., 2018).

Typically, informal care is manifested in the provision of personal care, which includes support in activities of daily living (ADL) such as eating, bathing, and toileting, and assistance in instrumental activities of daily living (IADL), such as household chores and transportation (e.g., Roth et al., 2015; Vergauwen & Mortelmans, 2020). The vast majority of the literature deals with the deleterious effects of caregiving on caregivers, including burden, burnout, depressive symptoms, and poor health (Bauer & Sousa-Poza, 2015; Lindt et al., 2020; Pinquart & Sörensen, 2003), although studies have also pointed to some positive aspects and psychological rewards (Di Novi et al., 2015; Roth et al., 2015). Yet, the mechanism of engaging in this complex and highly demanding role is still not fully understood (Zarzycki & Morrison, 2021). The present study aimed to elaborate on multilevel correlates of the provision of informal care to parents in need.

### Theoretical Perspective: The Informal Care Model

The present study used the Informal Care Model (ICM) proposed by van Groenou and De Boer (2016) to explain the provision of support to older parents. The ICM, an integrative and multicomponent framework, provides insight into the process of informal care. The process begins when someone in the social network needs help, mainly as a result of deteriorated health. According to the ICM, providing support is affected by macro-, mezzo-, and micro-level factors. The current analysis addressed mainly the mezzo- and micro-level correlates.

The mezzo level consists of contextual factors and refers to complementary sources of assistance to the informal caregivers, such as siblings and other members of the social network. A recent study showed that relatives and nonkin social contacts play an important role as complementary sources of care (Jacobs et al., 2018). The present study focused on the structural (network size) and quality (degree of satisfaction) dimensions of the social network, which are recognized as key factors in fostering better health and well-being (Antonucci et al., 2014; Schwartz & Litwin, 2019). With respect to informal care, the role of the social network was studied mainly in its potential to alleviate caregivers’ burden and stress (Lindt et al., 2020; Xu et al., 2021). However, social network characteristics have received only limited attention as antecedents of the provision of support to parents.

The ICM emphasizes the centrality of the micro level by relating to individual dispositions (van Groenou & De Boer, 2016), consisting of individual and relational layers. The examination at the individual layer addresses the role of the caregiver’s gender, as there is widespread consensus that daughters are more likely to be informal caregivers. Other characteristics include available resources that may facilitate the provision of care, such as younger age, having a partner, better health, higher financial capacity, and geographic proximity to the parent (Igel et al., 2009; Lorca et al., 2015; Tur-Sinai et al., 2020).

One of the main aspects relating to the relational layer, according to the ICM, is the affective quality of the relationship with the care receiver. Empirical studies have stressed the important role of the quality of the child–parent relationship in predicting support to older parents. The quality of the relationship was operationalized across the studies as emotional closeness, considering the parent as a confidant, and sharing a high quality of dyadic communication as perceived by the adult child (Carpenter, 2001; Cicirelli, 1993; Katz et al., 2010; Klaus, 2009; Lorca et al., 2015; Schwarz & Trommsdorff, 2005).

The ICM provided a theoretical framework for empirical studies that investigated the process of provision of care in mixed samples consisting of various types of relationships such as partners, relatives, and friends (e.g., de Klerk et al., 2021; Raiber et al., 2022), but has not yet been applied to the adult child–parent relationship. Given the high percentage of support provided by adult children, examining a multidimensional set of predictors of ICM can explain the complex nature of providing support to a parent. Another goal of the present study was to deepen the understanding of relationship-specific factors, which may serve as prerequisites for providing support to parents.

### Child–Parent Relationship Characteristics in the Provision of Support to Parents in Need

The complex mechanism of providing support to older parents is affected by the past history of the child–parent relationship and by whether the recipient of informal care is the mother or the father. These parameters received only limited research attention. According to the life-course perspective, experiences and conditions in older age are interrelated with earlier periods in life, presenting a cumulative learning history (Kesslet et al., 2014**).** The early child–parent relationship is the first learning environment in which social and mental development takes place (Elder, 1994). Empirical evidence shows that a better quality of relationship with the parents and parental emotional support in childhood are related to mental and physical well-being in later life (Damri & Litwin, 2019; Shaw et al., 2004).

From the perspective of attachment theory, the child’s early experiences of a sensitive and supportive parent who served as a secure base in times of distress are later internalized into a working model that enables higher social competence and better interpersonal relationships in adulthood (Ainsworth et al., 2015; Bowlby, 1969). In his pioneering work, Cicirelli (1993) argued that primary attachment is continuous and maintained into adulthood by seeking security and emotional closeness with the parent. Thus, the dependence of the parent may elicit the adult child’s perceived threat of losing the parent and lead to caregiving behaviors to maintain the parent as a resource.

Most of the studies focused on the affective quality of the present child–parent relationships as a predictor of support for older parents (e.g., Katz et al., 2010; Schwarz & Trommsdorff, 2005). One study (Silverstein et al., 2002) found that the affective relationship with the parents perceived by young adults was related to providing support to their parents 27 years later, but the connection between the child–parent relationship in childhood and providing support to older parents is still overlooked (Kong et al., 2021).

By contrast, parental abuse experienced in childhood may lead to poorer social functioning and maladaptive coping behaviors (Bowlby, 1969; Riggs et al., 2010). There has been evidence showing that childhood experiences of maternal abuse were associated with a lower frequency of emotional support provided by adult children (Kong & Moorman, 2016). In an early study (Whitbeck et al., 1991), contemporary emotional closeness mediated the association between parental rejection during childhood and less-frequent support given to old parents. However, the links between early maltreatment experiences and provision of support to parents are not unequivocal. For example, Silverstein and colleagues (2002) found that adult children responded to their parents’ needs despite the poor quality of their earlier relationship. In another study, a relatively high percentage of adult children who experienced abusive maternal behaviors in childhood provided support to their older mothers (Kong & Moorman, 2016). These findings may reflect the complexity and ambivalence of the intergenerational child–parent relationship (Kong et al., 2021).

Provision of support to an older parent in need also depends on whether the care recipient is the mother or the father. Empirical evidence showed that old mothers receive more support from their children than old fathers do. A possible explanation is that mothers tend to invest more in family relationships, eliciting long-term reciprocity on the part of the child (Klaus, 2009; Silverstein et al., 2002). Another reason is that a closer and more stable relationship is established with mothers rather than with fathers (Szydlik, 2008). Positive and negative evaluations of early relationships as prerequisites for providing support to parents were investigated mainly in child–mother relationships (e.g., Carpenter, 2001; Kong & Moorman, 2016; Schwartz & Trommsdorff, 2005). We are not aware of studies investigating the quality of affective relationships in predicting support to mothers and fathers separately. Investigating mezzo- and micro-level predictors of informal care provision to mothers and fathers separately is an additional contribution of the current analysis.

### Study Hypotheses

**Figure 1.**
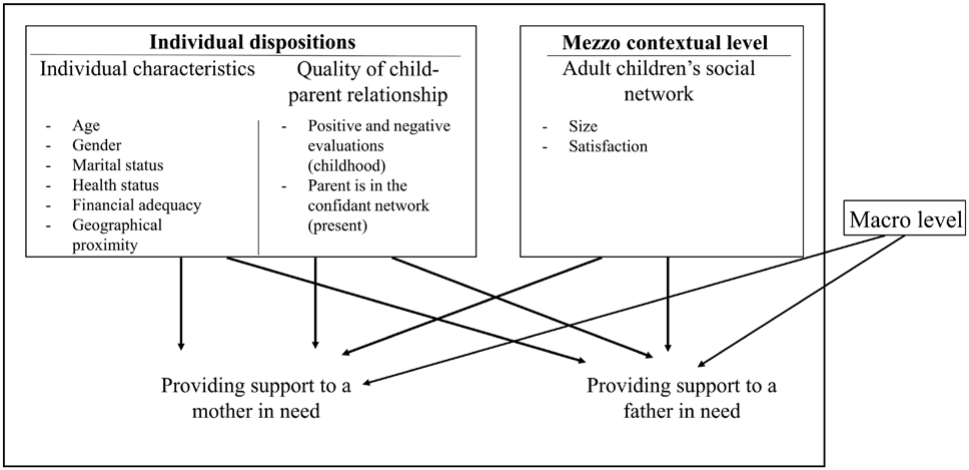
Research model.

Following the ICM, the present study is based on three sets of hypotheses about the mezzo and micro levels, controlling for the macro level (see Figure 1 for the research model). We examined the hypotheses separately for mothers and fathers in need. The first set of hypotheses is related to mezzo contextual factors and proposes that larger social network size and higher satisfaction from the social network are positively associated with provision of support. The second set of hypotheses focuses on individual characteristics and proposes that being female, younger, having a partner, better health, higher financial status, and closer geographic proximity to the parent predict providing support. The third set concerns child–parent relationship characteristics and includes the following hypotheses: Higher quality of affective relationship in childhood, lower frequency of parental abuse in childhood, and higher quality of affective relationship with the parent in the present are all related to provision of support. Because the limited support for the differences in the correlates depends on who the recipient of care is, we formulated the following research question: Are there differences in the correlates of provision of care based on the care recipient?

## Research Design, Methods, Data, and Sample

The data were derived from the Survey of Health, Ageing and Retirement in Europe (SHARE), a multidisciplinary panel survey covering 27 European countries and Israel. Community-dwelling adults aged 50 and older are biennially interviewed on various aspects of their lives, including health status, socioeconomic characteristics, and family and social networks (Börsch-Supan et al., 2013).

We used SHARE Waves 6 (2015), 7 (2017), and 8 (2019–2020). Wave 6 served as our baseline information, including individual background characteristics, present relationship with the parent, and respondents’ social network. Information on the relationship with the parent in childhood was derived from Wave 7, which included broad retrospective data. The outcome variable, provision of support to parents in need, was derived from Wave 8.

**Figure 2.**
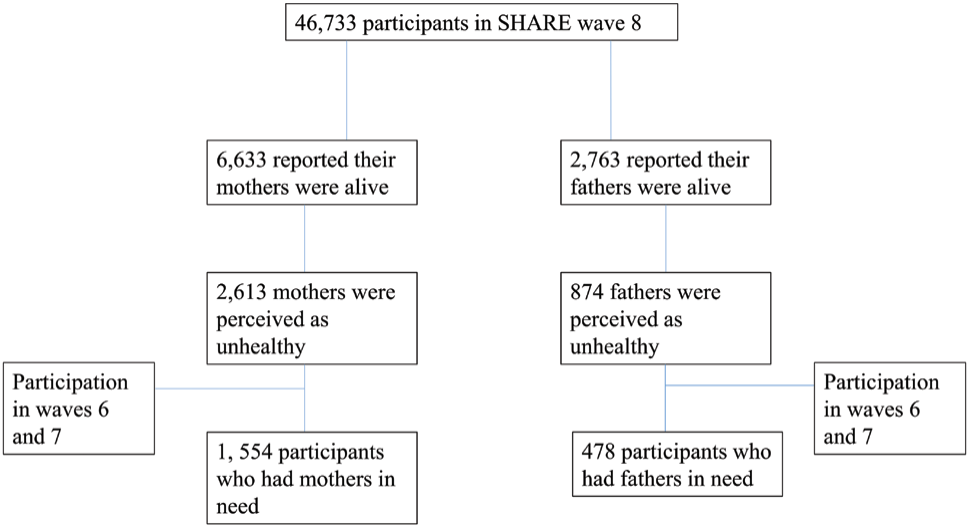
Flowchart for participant selection. SHARE = Survey of Health, Ageing and Retirement in Europe.

The current analytic sample comprised respondents who had a parent in need based on respondent perception in Wave 8. The evaluation was based on the parents’ health as perceived by respondents (0 = for a parent who was perceived as healthy [good to excellent health]; 1 = for an unhealthy parent [fair or poor health]). Included in the sample were respondents with a parent in need who also participated in Waves 6 and 7. To address differences based on the care recipient, the analytic sample comprised two subsamples: 478 respondents with a father and 1,554 respondents with a mother in need. See Figure 2 for further details on participant selection. Comparing the study sample with those reported having healthy parents showed that the included panel members were older and rated their health as poorer.

### Study Variables

#### Dependent variable: Providing support to a parent in need

Respondents were asked whether in the last 12 months, they have provided support in ADL (e.g., bathing and eating) and IADL (e.g., transportation, shopping, home repairs, and household chores) to a parent living outside the household. Support for a parent in the household referred only to assistance in ADL. We computed a binary variable, assigning a value of 0 if the respondent did not provide support in ADL or IADL and 1 if the respondent provided support. If the respondents reported having provided care, they were asked to whom, so the dependent variable was computed separately for mothers and fathers.

#### Independent variables

We operationalized **mezzo contextual factors** based on the *size* and *quality* of the respondents’ social network, which may include family members and nonkin. Respondents were asked to list up to seven persons, of whom up to six were considered to be their confidants (with whom they discussed important things), and the seventh was someone “who is very important to you for some other reason.” The respondents were also asked to rate their overall satisfaction with them on a scale of 0 (completely dissatisfied) to 10 (completely satisfied).

**Micro individual characteristics** included the respondents’ *age* (years), *gender* (0 for men, 1 for women), and *marital status,* dichotomized to living with a spouse or partner (1) or not (0). We addressed *health status* by two components: (a) self-rated physical health on a scale ranging from 1 (poor) to 5 (excellent) and (b) mental health, using depression assessed by the Euro-D scale (Prince et al., 1999). The scale assessed the number of depressive symptoms in the preceding month based on loss of appetite, lack of energy, and pessimism, ranging from 0 (not depressed) to 12 (very depressed). *Financial adequacy* was assessed by the perceived ability to make ends meet, ranging from 1 (with great difficulty) to 4 (easily). We measured *geographic proximity to the parent* separately for mother and father, ranging from 1 (living at a distance of 100 km or more from the parent) to 5 (living in the same household).

**Child–parent relationship characteristics** included two components: (a) quality of the relationship with the parent in *childhood,* captured through three questions assessed separately for mothers and fathers: (i) “How would you rate the relationship with your mother/father?” rated on a scale ranging from 1 (poor) to 5 (excellent); (ii) “How well did your mother/father understand your problems and worries?” rated on a scale ranging from 1 (not at all) to 4 (very well); and (iii) “How often did your mother/father push, grab, shove, throw something at you, slap, or hit you?” rated on a scale ranging between 1 (never) and 4 (often); and (b) the relationship with the parent in the *present,* assessed by the question: “Looking back over the past 12 months, who are the people with whom you most often discussed important things?” Listing the parent (mother and father separately) as part of the confidant network was coded 1 (0 otherwise).

The adjusted models controlled for the macro level by adding the geographic regions of residence: Northern Europe (Sweden, Denmark), Western Europe (Austria, Germany, France, Switzerland, Belgium, Luxembourg), Southern Europe (Spain, Italy, Greece, Israel, Croatia, Slovenia), Eastern Europe (Czech Republic, Poland), and the Baltic States (Estonia).

### Data Analysis

We present descriptive statistics of the study variables, followed by bivariate tests we conducted between each of the independent variables and the “provision of support to a parent” outcome. We conducted multivariate analyses using hierarchical logistic regression to examine three models: Model 1 concerned individual characteristics and available resources; Model 2 added early and contemporary child–parent relationship characteristics, containing positive and negative evaluations; and Model 3 focused on mezzo contextual factors containing information about the respondents’ social networks. The models also adjusted for the macro level using geographic regions. Additionally, we conducted a dominance analysis to address the unique effect of each variable in combination with the other variables in the regression (Kamin & Lang, 2016). We performed all the analyses separately for mothers and fathers in need. We used STATA 14 for the analysis.

## Results

The two subsamples comprised (1) respondents who had a mother in need (*n* = 1,544), of whom 37.13% (*n* = 886) provided support; and (2) respondents who reported having a father in need (*n* = 478), of whom 28.87% (*n* = 138) provided support.

We found similarities in the individual characteristics of the two subsamples. Almost 60% of respondents in both subsamples were women. Their mean age was 57.39 (standard deviation [*SD*] = 5.02) and 55.79 (*SD* = 4.68) for mothers and fathers in need, respectively. The majority reported living with a partner (77.35% [mothers] and 76.36% [fathers]). Average self-rated health was between “good” and “very good” (*M =* 3.20, *SD* = 0.97 [mothers] and *M =* 3.37, *SD* = 0.95 [fathers]), with few depressive symptoms (*M* = 2.22, *SD* = 2.05 [mothers] and *M* = 2.17, *SD* = 2.10 [fathers]). On average, respondents rated their perceived ability to make ends meet as “fairly easily” (*M* = 2.94, *SD* = 1.01 [mothers], and *M* = 3.03, *SD* = 0.99 [fathers]).

We also found similarities in the childhood relationship variables between the two subsamples. Respondents evaluated their relationship with mothers and fathers in childhood as good and higher (*M* = 3.65, *SD* = 1.03 [mothers], and *M* = 3.03, *SD* = 0.99 [fathers]). The perceived degree of mothers’ understanding was rated as “some understanding” (*M =* 3.05, *SD* = 0.92), slightly lower for fathers (*M =* 2.75, *SD* = 0.98). Regarding the present quality of the relationship with the parent, 27.09% of the respondents considered their mothers as a confidant and 16.9% considered their fathers to be part of their confidant network.

Respondents of both subsamples reported a low frequency of parent physical abuse in childhood (*M* = 1.63, *SD* = 0.86 [mothers], and *M* = 1.61, *SD* = 0.82 [fathers]); 18.28% and 16.53% reported experiencing parental abuse often or sometimes by mothers and fathers, respectively. Concerning mezzo contextual factors, respondents of both subsamples reported having approximately three confidants and very high overall satisfaction with their network (*M* = 8.99, *SD* = 1.13 [mothers], and *M* = 9.07, *SD* = 1.06 [fathers]). For further details of descriptive statistics, see Supplementary Table 1.

Table 1 presents bivariate associations between the three sets of predictors and the provision of support outcomes in both subsamples. The analyses showed that living closer to the parent and having higher financial adequacy were significantly related to supporting a parent in need (odds ratio [OR] = 1 .50, *p* < .01; OR = 1.28, *p* < .01 for mothers, OR = 1.70, *p* < .01; OR = 1.48, *p* < .01 for fathers), but being a woman and reporting better overall health were significant only in relation to supporting mothers (OR = 1.99, *p* < .01; OR = 1.16, *p* < .01).

**Table 1.** Bivariate Analyses of the Independent Variables With the Providing Support to a Parent in Need

Variable	Mother in need (*n* = 1,554)	Father in need (*n* = 478)
	OR	OR
Individual characteristics		
Women	1.99***	1.25
Age	1.00	1.02
Subjective health	1.16***	1.04
Depression symptoms	1.01	0.97
Living with a partner	0.79*	0.70*
Financial adequacy	1.28***	1.48***
Geographic proximity to parent	1.50***	1.70***
Child–parent relationship characteristics		
Relationship in childhood	1.10*	1.08
Degree of parent’s understanding in childhood	1.21***	1.09
Parent’s frequency of physical abuse in childhood	0.90	0.81*
Parent is in the confidant social network (present)	1.86***	1.68**
Mezzo contextual factors		
Size of the confidant social network	1.18***	1.17***
Satisfaction from the confidant social network	0.90	1.04

^a^Reference category—gender: men; marital status: living as single; parent in the confidant social network: parent is not in the confidant social network. OR = odds ratio.

***p < .01.

**p < .05.

*p < .1.

We found different patterns of child–parent relationships regarding mothers and fathers in need. Higher degree of mother’s understanding in childhood and her inclusion in the confidant social network at present were significantly related to providing support to her (OR = 1.21, *p* < .01; OR = 1.86, *p* < .01), but for fathers, only reporting on the inclusion of the father in the confidant social network in the present was significant (OR = 1.68, *p* < .05). Concerning the mezzo contextual factors, larger size of the confidant network was significantly related to providing support in both subsamples (OR = 1.18, *p* < .01 for mothers, OR = 1.17, *p* < .01; for fathers), but not the degree of satisfaction with the social network. The bivariate analysis between the predictor variables (Supplementary Tables 2 and 3) showed that in both subsamples, the strongest association was between the quality of relationship with the parents in childhood and the level of understanding the children received from the parents in childhood. In other words, when the children felt understood in childhood, they tended to rate the overall quality of their relationship with their parents in childhood higher.

In the multivariate analyses (Tables 2 and 3), we tested three models with individual characteristics (Model 1), child–parent relationship characteristics (Model 2), and mezzo contextual factors (Model 3). For the subsample that reported having a mother in need, Model 1 shows that being a woman, having higher financial adequacy, and living closer to the mother were related to increased odds of supporting her (OR = 2.15, *p* < .01; OR = 1.21, *p* < .01; OR = 1.68, *p* < .01). These findings remained significant after adjusting for relationship characteristics (Model 2) and mezzo contextual factors (Model 3). In child–parent relationship characteristics, higher degree of mother’s understanding in childhood and her inclusion in the confidant network at present predicted higher odds of supporting her (OR = 1.30, *p* < .01; OR = 1.59, *p* < .01). These patterns remained significant when adjusting for the mezzo contextual factors (Model 3). Finally, Model 3 shows that a larger confidant network was related to providing support (OR = 1.10, *p* < .05). Additionally, we performed the analyses with reference to the change in respondents’ network between Wave 6 and Wave 8, and found that a larger social network was borderline significant in predicting provision of support to mothers in need (OR = 1.28, *p* = .07).

**Table 2.** Odds Ratio Estimated for Individual Characteristics, Relationship With the Mother and Mezzo Contextual Factors, on Supporting Mothers in Need: Hierarchical Logistic Regression

Variable	Model 1^a^	Model 2^a^	Model 3^a^
	OR	OR	OR
Individual characteristics			
Women	2.15***	2.12***	2.07***
Age	1.01	1.02	1.01
Subjective health	1.14**	1.16**	1.14**
Depressive symptoms	1.05	1.05	1.04
Living with a partner	0.86	0.89	0.89
Financial adequacy	1.21***	1.20***	1.19***
Geographic proximity to mother	1.68***	1.65***	1.67***
Relationship with mother characteristics			
Relationship in childhood		0.90	0.91
Degree of mother’s understanding in childhood		1.30***	1.31***
Mother’s frequency of physical abuse in childhood		0.97	0.97
Mother is in the confidant social network (present)		1.59***	1.40**
Mezzo contextual factors			
Size of the confidant social network			1.10**
Satisfaction with the confidant social network			0.93
Pseudo *R*-squared	0.094	0.106	0.110
Δ*R*^2^		0.01***	0.004***
Observations	1,554	1,554	1,554

^a^Adjusted for geographic regions in Europe. OR = odds ratio.

***p < .01.

**p < .05.

*p < .1.

**Table 3.** Odds Ratio Estimated for Individual Characteristics, Relationship With the Father and Mezzo Contextual Factors, on Supporting Fathers in Need: Hierarchical Logistic Regression

Variable	Model 1^a^	Model 2^a^	Model 3^a^
	OR	OR	OR
Individual dispositions: background characteristics			
Women	1.50*	1.50	1.29
Age	1.03	1.03	1.03
Subjective health	1.02	1.02	1.01
Depressive symptoms	0.99	0.99	0.98
Living with a partner	0.74	0.78	0.78
Financial adequacy	1.58***	1.57***	1.57***
Geographic proximity to father	1.89***	1.89***	1.98***
Individual dispositions: relationship with father			
Relationship in childhood		0.94	0.94
Degree of father’s understanding in childhood		1.05	1.02
Father’s frequency of physical abuse in childhood		0.76*	0.74**
Father is in the confidant social network (present)		1.56	1.24
Mezzo contextual factors			
Size of the confidant social network			1.19**
Satisfaction with the confidant social network			1.03
Pseudo *R*-squared	0.106	0.118	0.128
Δ*R*^2^		0.01	0.01*
Observations	478	478	478

^a^Adjusted for geographic regions in Europe.

***p < .01.

**p < .05.

*p < .1

Concerning the relative contribution of each model in this subsample, Model 1 accounted for 9.42% of total outcome variance, Model 2 for 10.67%, and Model 3 for 11.04%. The relative contributions of Models 2 and 3 were found significant (Δ*R*^2^ = 0.01, *p* < .01; Δ*R*^2^ = 0.004, *p* < .01, respectively). Finally, dominance analysis revealed that the top three factors were geographic proximity to the mother, gender, and financial adequacy, accounting for about 50% of relative importance in explaining provision of support to mothers in need. These individual characteristics were followed by the inclusion of the mother in the confidant social network, the size of the social network, and the degree of mother’s understanding in childhood, with a standardized dominance of about 7%, 6%, and 4%, respectively.

A somewhat different picture was revealed regarding fathers. Model 1 shows that higher financial adequacy and living closer to the father were related to provision of support (OR = 1.58, *p* < .01; OR = 1.89, *p* < .01). These results remained significant after adjusting for the relationship characteristics (Model 2) and mezzo contextual factors (Model 3). The results of Model 2 show that the father’s physical abuse in childhood was borderline significant in negatively predicting provision of support (OR = 0.76, *p* = .07), and became statistically significant in Model 3, after adjusting for respondents’ mezzo contextual factors (OR = 0.74, *p* < .05). Thus, experiencing physical abuse from the father in childhood was related to lower odds of providing support to him. Finally, Model 3 shows that larger size of confidant network was related to provision of support (OR = 1.19, *p* < .05), but adding the change in respondents’ social network did not yield significant results. Regarding the relative contribution of the models, Model 1 accounted for 10.66 % of the total variance, Model 2 for 11.80%, and Model 3 for 12.84%. The differences between the models were nonsignificant. Dominance analysis revealed that the top two factors were geographic proximity to the father and financial adequacy, accounting for about 65% of the relative importance in explaining provision of support to fathers in need. The size of the respondents’ social network and the frequency of physical abuse by the father in childhood were ranked next, with a standardized dominance of about 9% and 5%, respectively.

## Discussion and Implications

The present study aimed to explore various correlates of provision of support to an older parent in need. The research was based on the ICM theoretical model, comprising mezzo contextual variables and micro-level variables consisting of individual and relational layers. We paid special attention to the child–parent relationship, assessing the quality of relationship in childhood and at present from the caregivers’ perspective, and relating to the care recipient.

We assessed the mezzo contextual factors based on the structural and quality dimensions of the social network. The results show that a larger network, expressed in a higher number of confidants listed by the adult children, was related to increased chances of providing support to both mothers and fathers in need. Overall degree of satisfaction with the social network was not significant, which is partially consistent with a recent study (Xu et al., 2021) reporting that structural aspects of the social network had a stronger buffer effect on informal caregivers’ burden than did satisfaction with the social support. Our finding may be explained by the measure used to assess the structural aspect of the network, which asked about the number of confidants and therefore reflected more on the quality of the relationships. Evidence of that was provided by the very high reported satisfaction with the network.

The micro-level correlates included the respondents’ background characteristics and available resources. We found higher rates of support provided to mothers than to fathers. This is consistent with the demographic trend of older men usually being supported by their spouses, whereas older women (more frequently widowed) are supported mostly by their adult children (Li et al., 2022). The study showed that being a daughter predicted providing support only to mothers in need. This finding is consistent with the demographic trend showing that adult daughters and older mothers are the most prevalent intergenerational caregiving dyad (Coe et al., 2018). It may also indicate the importance of intergenerational relationships between women. Mothers tend to invest more and receive more support, mainly from daughters (Igel, 2009; Szydlik, 2008).

The findings also indicate that in both subsamples, geographic proximity and higher financial adequacy were the strongest predictors of provision of support to a parent in need, as reported in previous studies (e.g., Herrera & Fernández, 2022; Katz et al., 2010). A weaker predictor was the adult child’s health status. Better perceived health was related to providing support to mother but not to father. This finding reflects and may explain the inconsistency in previous studies, some of which found that better health correlated with the provision of more informal care (Bauer & Sousa-Poza, 2015; Igel et al., 2009), and others reported no such association (Lorca et al., 2015; Tur-Sinai et al., 2020). A possible explanation for not finding a significant association between the health of the provider and the provision of care to fathers is that older men usually rely on their wives for support, who tend not to share with others the caregiving burden (Li et al., 2022). In the absence of a spouse, supporting may emerge as an obligation, restricting the adult children’s choices. Therefore, support may be extended to older fathers irrespective of the adult children’s health status.

The main focus of the present study was the role of the quality of child–parent relationships in the provision of support. The findings show that positive evaluations of the relationship with mothers were related to increased odds of providing support for her. The relationship included the degree of maternal understanding of the participants’ worries and problems in childhood, and the inclusion of the mother in the confidant network at present. The findings are consistent with those of previous studies that emphasized the important role of emotional bonds with mothers in shaping adult children’s caregiving behavior (Carpenter, 2001; Schwarz & Trommsdorf, 2005). This caregiving behavior may be rooted in early childhood. Mothers are usually the primary caregivers, which may elicit a higher attachment of the children to them (Klaus, 2009). Following Cicirelli’s (1993) argument, caregiving behaviors may be interpreted as an attempt to preserve the mothers as a resource and delay their loss.

We found an opposite pattern with regard to fathers in need. Only the negative evaluation of the relationship, measured by physical abuse in childhood, was related to reduced odds of provision of support. This finding is consistent with those of studies that found associations between parental maltreatment and lower frequency of care in later life (Kong & Moorman, 2016; Whitbeck et al., 1991).

The evidence that maternal abuse was not related to the provision of support whereas abuse was the only relationship marker related to the provision of support to fathers is intriguing, especially given the similar frequency of parental physical abuse by fathers and by mothers (around 17%). A possible explanation may be the cardinal effect of early attachment which has been shown to be stronger with mothers than with fathers, and its effect on providing care remains even despite early maternal abuse (Kong et al., 2021). This notion was reinforced by Kong and Martire’s findings (2019) that the quality of contemporary relationships with mothers, but not fathers, mediated the association between maternal childhood abuse and psychological well-being in later life.

Finally, the present study used a multidimensional approach to explore factors related to providing support to a parent in need. Consistent with the ICM, we included mezzo contextual, individual, and child–parent relationship factors in the analyses, controlling for the macro level. The findings revealed that the dominant group of predictors was that of individual characteristics and personal resources. This finding is consistent with Szydlik’s conclusion (2008) that “who has more gives more,” and stresses the importance of adult children’s resources in shaping their caregiving behavior toward their parents (Camden et al., 2011; Herrera & Fernández, 2022).

The study has several limitations. Despite its longitudinal design, it cannot rule out that reciprocal associations may exist between individual dispositions, relationship factors, contextual factors, and supporting parents in need. For example, the size of the social network may not only positively influence the provision of support, but adult children may enlarge their social network as a coping strategy for the burdensome aspects of caregiving. Another limitation is that the macro level, which may also affect the provision of support to parents, received only limited attention. It has been shown that determinants and characteristics of informal care vary across different welfare regimes of formal care and family orientation in Europe (Katz et al., 2010; Verbakel, 2018). The present study controlled for geographic regions to gain deeper insights into mezzo- and micro-level factors, specifically the quality of the child–parent relationship in childhood and at present.

Future studies should use a macro–mezzo–micro model and examine reciprocal links in these dimensions more thoroughly. Given the increased need for care of older individuals, the study findings suggest that to facilitate and preserve the provision of support to older parents in need, efforts should be made to increase adult children’s individual resources. These include, among others, support at the workplace with shorter work days for informal caregivers, tax reductions, and career benefits. Given the important role of the quality of the relationship with the parent in childhood and at present in the provision of care, clinical efforts should be focused on improving this relationship to increase the emotional closeness between the parent and the adult child, and if needed, to address unresolved emotional issues with the parent (Kong et al., 2021). Clinicians should also be aware that when the father is in need of care, abusive behavior in childhood jeopardizes the ability of the grown-up child to provide it. The results also point to the importance of the confidant social network, which may consist of nonkin relationships; therefore, efforts should be made to increase the close relationships of the care provider. These may include social support platforms that provide emotional support by health care professionals and enable interactions with other informal caregivers going through similar experiences (Sitges-Maciá et al., 2021). Based on our findings, policy-makers should increase the resources available to caregivers and support interventions aimed at advancing close social and family relationships to encourage the provision of care to older adults in need.

## Supplementary Material

igad032_suppl_Supplementary_Material

## Data Availability

This paper uses data from SHARE Waves 6, 7, and 8 (dois: 10.6103/SHARE.w6.800, 10.6103/SHARE.w7.800, 10.6103/SHARE.w8.800, 10.6103/SHARE.w8ca.800); see Börsch-Supan et al. (2013) for methodological details. The SHARE data are available to the research community upon individual registration: http://www.share-project.org/data-access/user-registration.html
