# Supplementary material for: Support for Older Parents in Need in Europe: The Role of the Social Network and of Individual and Relational Characteristics
Source: Innov Aging. 2023 Apr 17;7(4):igad032. doi: 10.1093/geroni/igad032 (PMC10195565; doi:10.1093/geroni/igad032)
Supplement: igad032_suppl_Supplementary_Material [file igad032_suppl_supplementary_material.docx]

**Online Supplementary Material**

**Supplementary Table 1**

*Descriptive statistics of the study variables: Individual and child-parent relationship characteristics, mezzo contextual and macro factors*

|  | Mother in need  (*n* = 1,554) | Father in need  (*n* = 478) |  |
| --- | --- | --- | --- |
|  | Mean (SD)/n (%) | Mean (SD)/ n (%) | Range |
| **Dependent variable** |  |  |  |
| Providing support to a parent in need | 886 (37.13%( | 138 (28.87%) |  |
| **Individual characteristics** |  |  |  |
| Women | 931 (59.91%) | 59.21% |  |
| Age | 57.39 (5.02) | 55.79 (4.68) |  |
| Subjective health | 3.20 (0.97) | 3.37 (0.95) | 1-5 |
| Depressive symptoms | 2.22 (2.05) | 2.17 (2.10) | 0-12 |
| Living with a partner | 1202 (77.35%) | 365 (76.36%) |  |
| Financial adequacy | 2.94 (1.01) | 3.03 (0.99) | 1-4 |
| Geographic proximity to parent | 2.78 (1.09) | 2.59 (1.06) | 1-5 |
| **Child-parent relationship characteristics** |  |  |  |
| Relationship in childhood with parent | 3.65 (1.03) | 3.42 (1.09) | 1-5 |
| Degree of parent's understanding in childhood | 3.05 (0.92) | 2.75 (0.98) | 1-4 |
| Parent's frequency of physical abuse in childhood | 1.63 (0.86) | 1.61 (0.82) | 1-4 |
| Parent is in the confidant social network (present) | 421 (27.09%) | 81(16.95%) |  |
| **Mezzo contextual factors** |  |  |  |
| Size of the confidant social network | 2.94 (1.58) | 3.09 (1.64) | 0-7 |
| Satisfaction with the confidant social network | 8.99 (1.13) | 9.07 (1.06) | 1-10 |
| **Macro level: Geographic regions** |  |  |  |
| North | 183 (11.77%) | 69 (14.44%) |  |
| West | 685 (44.08%) | 232 (48.54%) |  |
| East | 87 (5.59%) | 26 (5.44%) |  |
| South | 467 (30.05%) | 133 (27.82%) |  |
| Baltic | 132 (8.49%) | 18 (3.77%) |  |

^a^ Reference category- providing support to a parent in need: not providing support; gender: men; marital status: living as single; parent in the confidant social network: parent is not in the confidant social network.

**Supplementary Table 2**

*Pearson correlation of independent variables in the subsample of mothers in need (n-=1,554)*

| Variables | (1) | (2) | (3) | (4) | (5) | (6) | (7) | (8) | (9) |
| --- | --- | --- | --- | --- | --- | --- | --- | --- | --- |
| (1) Age |  |  |  |  |  |  |  |  |  |
| (2) Subjective health | -0.104*** |  |  |  |  |  |  |  |  |
| (3) Depressive symptoms | -0.058** | -0.371*** |  |  |  |  |  |  |  |
| (4) Financial adequacy | 0.067*** | 0.221*** | -0.200*** |  |  |  |  |  |  |
| (5) Geographical proximity | 0.012 | -0.035 | -0.028 | -0.102*** |  |  |  |  |  |
| (6) Relationship in childhood with mother | -0.026 | 0.081*** | -0.125*** | 0.009 | 0.110*** |  |  |  |  |
| (7) Degree of mother’s understanding in childhood | -0.010 | -0.002 | -0.073*** | 0.005 | 0.106*** | 0.670*** |  |  |  |
| (8) Mother’s physical abuse in childhood | 0.019 | -0.068*** | 0.089*** | -0.049 | -0.032 | -0.260*** | -0.259*** |  |  |
| (9) size of the confidant social network | -0.004 | 0.098*** | 0.002 | 0.124*** | -0.055** | 0.019 | 0.021 | -0.001 |  |
| (10) Satisfaction with the confidant social network | -0.031 | 0.076*** | -0.146*** | 0.022 | 0.030 | 0.174*** | 0.114*** | -0.030 | -0.010 |

** p<0.1, ** p<0.05, *** p<0.01*

**Supplementary Table 3**

*Pearson correlation of independent variables in the subsample of fathers in need (n-=478)*

| Variables | (1) | (2) | (3) | (4) | (5) | (6) | (7) | (8) | (9) |
| --- | --- | --- | --- | --- | --- | --- | --- | --- | --- |
| (1) Age |  |  |  |  |  |  |  |  |  |
| (2) Subjective health | -0.112** |  |  |  |  |  |  |  |  |
| (3) Depressive symptoms | 0.018 | -0.377*** |  |  |  |  |  |  |  |
| (4) Financial adequacy | 0.075 | 0.116** | -0.235*** |  |  |  |  |  |  |
| (5) Geographical proximity | 0.068 | -0.065 | 0.008 | -0.089* |  |  |  |  |  |
| (6) Relationship in childhood with father | -0.021 | 0.115** | -0.110** | 0.013 | 0.012 |  |  |  |  |
| (7) Degree of father’s understanding in childhood | 0.029 | 0.059 | -0.141*** | -0.002 | 0.031 | 0.688*** |  |  |  |
| (8) Father’s physical abuse in childhood | 0.106** | -0.031 | -0.005 | 0.040 | -0.010 | -0.400*** | -0.307*** |  |  |
| (9) size of confidant social network | 0.048 | 0.026 | 0.057 | 0.096** | -0.119*** | 0.071 | 0.091* | 0.014 |  |
| (10) Satisfaction with the confidant social network | -0.046 | 0.128*** | -0.208*** | -0.052 | 0.028 | 0.150*** | 0.114** | -0.061 | -0.050 |

** p<0.1, ** p<0.05, *** p<0.01*
